# Comparison of Prediction Model for Cardiovascular Autonomic Dysfunction Using Artificial Neural Network and Logistic Regression Analysis

**DOI:** 10.1371/journal.pone.0070571

**Published:** 2013-08-05

**Authors:** Zi-Hui Tang, Juanmei Liu, Fangfang Zeng, Zhongtao Li, Xiaoling Yu, Linuo Zhou

**Affiliations:** 1 Department of Endocrinology and Metabolism, Fudan University Huashan Hospital, Shanghai, China; 2 Department of Computer Science, Youzhou Vocational and Technology Collage, Yongzhou, Hunan, China; Cardiff University, United Kingdom

## Abstract

**Background:**

This study aimed to develop the artificial neural network (ANN) and multivariable logistic regression (LR) analyses for prediction modeling of cardiovascular autonomic (CA) dysfunction in the general population, and compare the prediction models using the two approaches.

**Methods and Materials:**

We analyzed a previous dataset based on a Chinese population sample consisting of 2,092 individuals aged 30–80 years. The prediction models were derived from an exploratory set using ANN and LR analysis, and were tested in the validation set. Performances of these prediction models were then compared.

**Results:**

Univariate analysis indicated that 14 risk factors showed statistically significant association with the prevalence of CA dysfunction (P<0.05). The mean area under the receiver-operating curve was 0.758 (95% CI 0.724–0.793) for LR and 0.762 (95% CI 0.732–0.793) for ANN analysis, but noninferiority result was found (P<0.001). The similar results were found in comparisons of sensitivity, specificity, and predictive values in the prediction models between the LR and ANN analyses.

**Conclusion:**

The prediction models for CA dysfunction were developed using ANN and LR. ANN and LR are two effective tools for developing prediction models based on our dataset.

## Introduction

The prevalence of cardiovascular autonomic (CA) dysfunction is rapidly increasing worldwide, particularly in developing countries. The disease is not only a major factor in the cardiovascular complications of diabetes mellitus (DM) [1], but it also affects many other major segments of the general population, such as the elderly and patients with hypertension (PH), metabolic syndrome (MetS), and connective tissue disorders [2]–[4]. CA dysfunction has become a major health concern in China following rapid changes in lifestyle. The prevalence of CA dysfunction in diabetic patients was found to be 30–60% [1]. Individuals with previously undiagnosed CA dysfunction have an unfavorable cardiovascular risk profile, especially the risk of sudden death, indicating a higher risk for cardiovascular disease [5]. CA function testing using HRV is sensitive, noninvasive, and reproducible; therefore, it is easily applicable for screening a large number of individuals in the general population [6].

In clinical medicine, a prediction model refers to the type of medical research study using which researchers try to identify the best combination of medical signs, symptoms, and other findings that may be used to predict the probability of a specific disease or outcome [7]. These models may aid the clinician in the decision-making process regarding clinical admission, early prevention, early clinical diagnosis, and application of clinical therapies. Most previous prediction models were developed using univariate or multivariate logistic regression (LR) analysis [8], [9]. An artificial neural network (ANN) refers to a mathematical model inspired by biological neural networks [10]. ANNs consist of an interconnected group of artificial neurons, and they process information using a connectionist approach to computation. ANNs employ nonlinear mathematical models to mimic the human brain’s own problem-solving process, by using previously solved examples to build a system of “neurons” that makes new decisions, classifications, and forecasts [11]. ANN is a complex and flexible nonlinear system with exclusive properties consisting of robust performance in dealing with noisy or incomplete input patterns, high fault tolerance, and the ability to make generalizations on the basis of the input data. ANN is often applied to model complex relationships between inputs and outputs or to find patterns in data. In clinical medicine, ANN models have been applied in the diagnosis of diseases such as myocardial infarction [12]. ANN models have also been successfully used to predict trauma mortality and in clinical decision-making in the management of traumatic brain injury patients [13], [14]. A previous study compared the LR and ANN models used in the prediction of living setting after hip fracture [15].

Thus far, no studies in literature have used ANN for modeling CA dysfunction prevalence in the general population instead of diabetic patients. The aim of this study was to develop prediction models for CA dysfunction using ANN and LR. In addition, we compare prediction models built by the two approaches. The ANN model is at least as accurate as the LR model for the prediction of outcomes from our dataset.

## Materials and Methods

### Study Population

We analyzed a previously constructed database of a CA dysfunction survey carried out in a random sample of middle-aged Chinese individuals. Participants were recruited from three communities in Shanghai, China, primarily from the Baoshan District area. Participants with undiagnosed CA dysfunction, aged 30–80 years, were included in this study. A total of 3,012 subjects were invited to a screening visit between 2011 and 2012. Subjects with potential confounding factors that may influence cardiac autonomic function were excluded from the study. The exclusion criteria were as follows: (a) history or findings of arrhythmia (i.e., heart block, atrial fibrillation, ventricular tachycardia), hyperthyroidism or hypothyroidism, and dilated or hypertrophic cardiomyopathy; (b) pregnancy or lactation; and/or (c) a major systemic illness such as systemic lupus erythematosus. A total of 2,092 (69.46%) participants with complete baseline data were obtained. Written consent forms were obtained from all the patients before the start of the study. The study protocol was approved by the Ethics Committee of Huashan Hospital, Shanghai, China. The subjects were interviewed to document their medical histories and medication, history of smoking habits, laboratory assessment of cardiovascular disease risk factors, and standardized examination for HRV. All study subjects underwent a complete CAF evaluation after fasting for eight hours. The evaluation included: (a) history and physical examination, (b) heart rate and blood pressure, (c) fasting serum glucose and insulin, and (d) fasting plasma lipids. The body mass index was calculated as the weight in kilograms divided by the square of the height in meters.

Systolic and diastolic blood pressure (BP) values were the means of two measurements obtained by the physician on the left arm of the seated participant. Fasting plasma glucose (FPG) was quantified by the glucose oxidase procedure, and HbA1c was measured by ion-exchange high-performance liquid chromatography (HPLC; Bio-Rad, Hercules, CA, USA). The homeostasis model assessment insulin resistance estimate (HOMA-IR) was calculated as serum glucose (mmol/L) multiplied by plasma insulin (mU/mL) divided by 22.5, and the serum total cholesterol (TC), high-density lipoprotein (HDL) cholesterol, triglyceride (TG) levels, creatinine (Cr), and uric acid (UA) levels were measured enzymatically with a chemical analyzer (Hitachi 7600-020, Tokyo, Japan). Low-density lipoprotein (LDL)-cholesterol levels were calculated using the Friedewald formula, and the creatinine clearance rate (Ccr) was calculated using the Cockcroft-Gault formula. The day-to-day and inter-assay coefficients of variation at the central laboratory in our hospital for all analyses were between 1% and 3%. Short-term HRV test was applied to evaluate CA function. HRV was measured non-invasively by power spectral analysis. Subjects were studied while awake and in the supine position after 20 minutes of rest. Testing times were from 8∶00 AM to 11∶00 AM, and 1∶30 PM to 4∶30 PM. A type-I FDP-1 HRV BRS non-invasive detection system was used (version 2.0; Department of Biomedical Engineering, Fudan University, Shanghai, China). Electrocardiography and respiratory signals and beat-to-beat blood pressure were continually and simultaneously recorded for 15 minutes by using an electrosphygmograph transducer (HMX-3C) placed on the radial artery of the dominant arm and an instrument respiration sensor. Short-term HRV analysis was performed for all the subjects using a computer-aided examination and evaluation system for spectral analysis to investigate changes in autonomic regulation.

### Definition

PH was defined as blood pressure ≥140/90 mmHg or history of anti-hypertensive medication. BMI was classified on the basis of Chinese criteria: normal, <24.0 kg/m^2^; overweight, ≥24.0 kg/m^2^<28.0 kg/m^2^; obese, BMI ≤28.0 kg/m^2^. Fasting plasma glucose (FPG) levels ≥5.6 mmol/L were considered high. Central obesity was defined using ethnicity-specific values: waist circumference (WC) ≥90 cm in men or ≥80 cm in women [16]. Serum triglyceride (TG) levels ≥1.7 mmol/L were considered high. Serum high-density lipoprotein-cholesterol (HDL-C) levels <0.9 mmol/L in men or <1.0 mmol/L in women were considered low. Diabetes was diagnosed by the oral glucose tolerance test (OGTT) and determined by either HbAlc ≥6.5% or the use of insulin or hypoglycemic medications. Individuals meeting three or more of the updated National Cholesterol Education Program/Adult Treatment Panel III criteria (WHO Western Pacific Region obesity criteria) were diagnosed as having MetS [16]. CAN was diagnosed on the basis of at least two abnormal cardiovascular autonomic reflex test results [1].

### Statistical Analysis

The Kolmogorov-Smirnov test was used to determine whether continuous variables followed a normal distribution. Variables that were not normally distributed were log-transformed to approximate normal distribution for analysis. The results are expressed as means ± standard deviation or medians, unless otherwise stated. The subject characteristics according to MetS severity scores were assessed using one-way analysis of variance (ANOVA) for continuous variables and the *χ* ^2^ test for categorical variables. Potential CA dysfunction risk factors, which are known clinically and in literature to be associated with CA dysfunction, were selected for the evaluation. These factors included age (sorted into three age groups: ≤50, 51–60, and >60 years), gender, BMI, abdominal obesity (WC ≥90 or ≥80 cm in men and women respectively), current smokers (yes/no), resting heart rate (HR; categorized into four groups: ≤70, 71–80, 81–90, and >90 beats/min), diabetes, hypertension, blood glucose profile, lipid profile, and renal profile. Univariate analyses were performed to estimate the significant predictors of CA dysfunction.

### Multivariate Logistic Regression Models

A computerized random number generator was used to select three-fourths of the patients to make up the exploratory set to develop prediction models. The remaining one-fourth of the patients comprised the validation set. These steps were repeated 5 times in order to generate five pairs of exploratory and validation sets which were saved for further processing by LR and ANN.

Multiple LR analysis was used to compute the *β* coefficients for known risk factors for CA dysfunction. To develop a good-fit model, all significant variables derived from univariate analysis were entered into the model. Variables significant at 5% were included in the multiple LR using stepwise backward elimination, with CA dysfunction as the dependent variable. The independent continuous variables of age and resting HR were categorized. A *P* value of ≤0.05 was considered significant. A prediction model was developed using the probability value calculated from summary score assigned to final variables based on its regression coefficient. The probability value was calculated for each participant. The performance of the prediction model developed in this study was evaluated in the validation set.

### Artificial Neural Network Models

**Figure 1:**
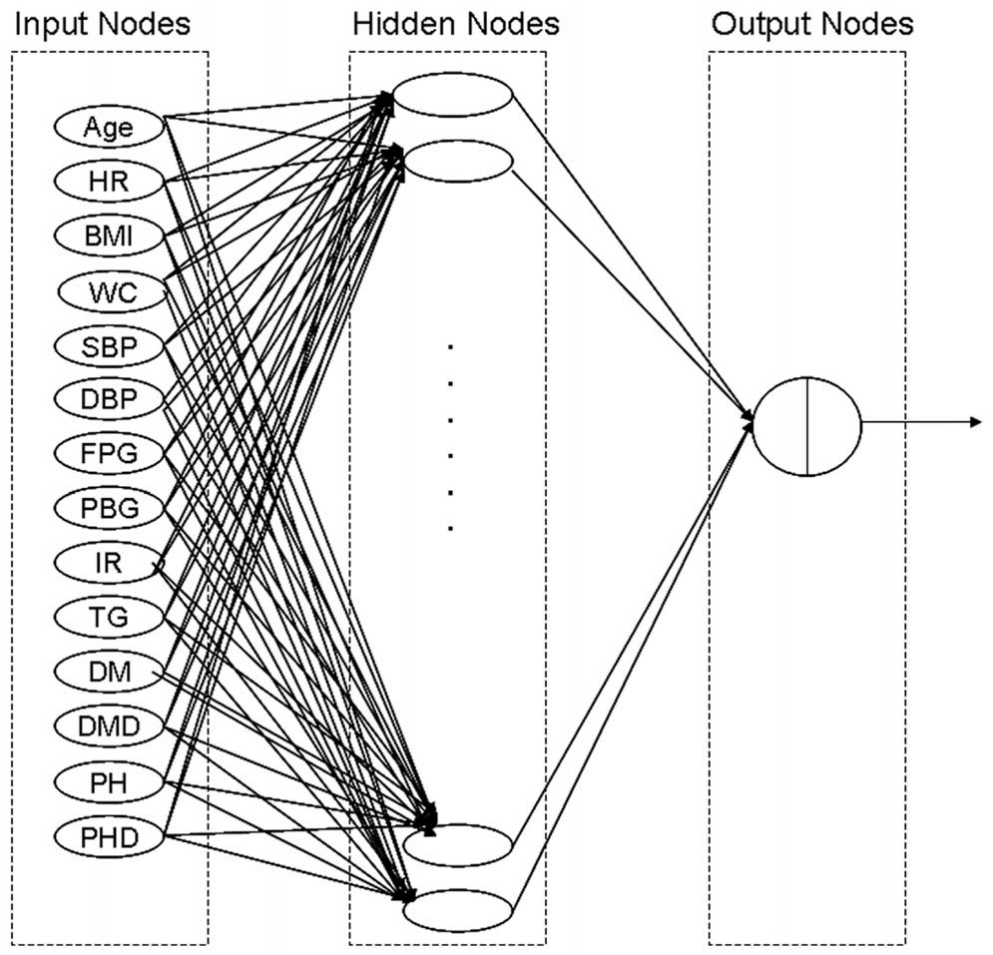
Artificial neural network model showing input variables (nodes), hidden nodes, and connection weights with output node for data on CA dysfunction. The ANN model including 14 input nodes, 18 hidden nodes and 1 output node. Data from a total of 2077 patients had been used to ANN analysis. BMI- Body mass index, WC-waist circumference, SBP- systolic blood pressure, DBP- diastolic blood pressure, FPG- fasting plasma glucose, PBG- plasma blood glucose, IR-insulin resistance, TG- triglyceride, UA- uric acid, HR-heart rate, PH- Hypertension, DM- Diabetes, PHD- Hypertension duration, DMD- Diabetes duration.

The ANN applied in this study was a standard feed-forward, back-propagation (BP) neural network with three layers consisting of an input layer, a hidden layer, and an output layer. The input layer contained 14 input neurons, the hidden layer contained 18 neurons, and the output layer contained 1 output neuron (Figure 1). The number of hidden layer neurons was determined through trial and error, since no accepted theory currently exists for predetermining the optimal number of hidden layer neurons [17]. The number of hidden layer neurons was selected to lead to a predictive network with the best sensitivity and specificity. The training datasets were the same as the exploratory sets used in the LR model. During the training, the learning rate (applies a greater or lesser portion of the respective adjustment to the old weight) and momentum for network training (basically allows a change to the weights to persist for a number of adjustment cycles) were set to 0.20 and 0.9, respectively. To obtain the connection weights, the network first underwent a training process using the BP of error method, which employs the generalized delta learning rule. This is an iterative process by which input derivation sets are used to the ANN, and outputs are calculated. The output is then compared to the desired output, and the connection weights are adjusted based on the error in output. A validation dataset was developed to avoid an over-fitting ANN model. In general, one-fourth of the patients were randomly selected from the exploratory set. The training was run until a minimum average square error (MSE) of <0.001 or an increasing MSE was found in the validation dataset. The performance of the prediction model was evaluated in testing datasets. The testing datasets were the same as the validation sets used in the LR model.

### Model Comparison

Discrimination and calibration were both measured. Discrimination refers to the ability of a model to distinguish between individuals with and without CA dysfunction. The discriminatory power of the models was analyzed using a receiver-operating characteristic (ROC) curve and area under the curve (AUC). ROC curves were constructed by plotting true positives versus the false positive fraction. Sensitivity (the probability of a positive test given the individual has the disease), specificity (the probability of a negative test given the individual does not have the disease), positive predictive value (the probability of having the disease given a positive test), and the negative predictive value (the probability of not having the disease given a negative test) were calculated for each cutoff score. The cutoff score that gave the maximum sum of sensitivity and specificity was considered optimum [18]. Calibration refers to how accurately the models predicted over the entire range. The calibration of models was computed using the Hosmer-Lemeshow (HL) test, which is a single summary measure of the calibration and is based on comparing the observed and estimated prevalence of disease grouped by estimated prevalence [19]. The HL statistic follows a χ^2^ distribution, with degrees of freedom equal to two less than the number of groups. The overall accuracy (ratio of summary of the number of true positive and true negative results to the total sample size) of the prediction model was calculated by comparing the predicted values with the actual events.

All parameters of discrimination were evaluated in the five validation sets. Prediction models from LR and ANN based on AUC were compared. The mean the AUC, sensitivity, specificity, and predictive values were calculated and compared with non-inferiority tests. Odds ratios (OR) with 95% confidence intervals (CI) were calculated for the relative risk of predictors with outcome. Results were analyzed using the Statistical Package for Social Sciences for Windows version 16.0 (SPSS; Chicago, IL, USA). The BP ANN models were developed using Matlab 7.0. A *P* value of <0.05 was considered significant.

## Results

The baseline clinical characteristics of the 2092 subjects were listed in Table 1. The entire sample included 705 men and 1387 women (mean age, 60.42±8.68 years; Table 1), of which 387 (18.51%) were found to have CA dysfunction. The mean BMI and WC were 24.21 kg/m^2^ and 85.07 cm, respectively. The HRV components decreased with age (data not shown). The HR of individuals with CA dysfunction was very significantly higher than that of individuals without CA dysfunction (P<0.001). Except for LF/HF, most HRV parameters were lower in individuals with CA dysfunction than in those without CA dysfunction (P<0.01 for all). The majority of subjects were not affected heart failure (99.07%), and the prevalence of PH, DM, and MetS in the entire sample was 46.65, 21.33, and 39.82%, respectively. The baseline characteristics were similar between the exploratory and validation sets (p<0.05 for all).

**Table 1:** Baseline characteristics of subject.

Variables	Individuals without CA dysfunction	Individuals with CA dysfunction	Entire sample	*P* value*
N	1705	387	2092	
Age	59.85±8.64	62.94±8.43	60.42±8.68	<0.001
Gender male,%	562 (32.96%)	143 (36.95%)	705 (33.7%)	0.134
Height cm	161.46±7.78	161.45±7.83	161.46±7.79	0.987
Weight kg	62.9±10.47	64.85±11.09	63.26±10.61	0.001
BMI kg/m^2^	24.07±3.26	24.84±3.69	24.21±3.36	<0.001
WC cm	84.48±9.54	87.68±9.93	85.07±9.70	<0.001
SBP mmHg	126.41±18.14	132.95±20.02	127.62±18.68	<0.001
DBP mmHg	79.50±9.61	81.28±9.93	79.83±9.69	0.001
Laboratory assays				
FPG mmol/L	5.4±1.57	6.12±2.53	5.53±1.81	<0.001
PBG mmol/L	7.36±3.22	9.03±4.53	7.67±3.56	<0.001
FINS IU/L	6.74±8.01	9.17±21.66	7.19±11.82	<0.001
IR	1.64±2.12	2.54±6.21	1.81±3.3	<0.001
TC mmol/L	5.31±0.98	5.39±1.05	5.32±1	0.142
TG mmol/L	1.67±0.92	1.9±1.17	1.71±0.98	<0.001
HDL mmol/L	1.36±0.33	1.34±0.32	1.36±0.32	0.203
LDL mmol/L	3.18±0.76	3.23±0.8	3.19±0.77	0.229
SCr µmol/L	77.65±26.89	78.51±21.93	77.81±26.04	0.561
Ccr	82.17±30.42	81.31±32.65	82.01±30.84	0.624
UA µmol/L	280.13±83.25	285.97±86.04	281.21±83.79	0.216
HRV measurement				
HR beats/min	70.77±9.08	79.7±11.26	72.42±10.13	<0.001
TP ms^2^	1000.63±693.2	315.87±410.75	873.95±702.47	<0.001
LF ms^2^	224.34±215.08	43.97±57.29	190.98±207.88	<0.001
LF nu	22.54±10.6	15.97±9.19	21.33±10.66	<0.001
HF ms^2^	215.11±229.61	41.82±59.63	183.05±219.43	<0.001
HF nu	21.49±12.94	17.06±13.98	20.67±13.25	<0.001
LF/HF	1.55±1.48	2.37±3.32	1.7±1.98	<0.001
Medical history				
Smoking yes,%	244 (14.31%)	62 (16.02%)	306 (14.63%)	0.390
PH yes,%	735 (43.11%)	241 (62.27%)	976 (46.65%)	<0.001
DM yes,%	307 (18.02%)	139 (35.92%)	446 (21.33%)	<0.001
MetS yes,%	629 (36.89%)	204 (52.71%)	833 (39.82%)	<0.001

Note: * present difference of baseline characteristics between individuals with and without CA dysfunction. BMI- Body mass index, WC-waist circumference, SBP- systolic blood pressure, DBP- diastolic blood pressure, FPG- fasting plasma glucose, PBG- plasma blood glucose, FINS- fasting blood insulin, IR-insulin resistance, TC- serum total cholesterol, TG- triglyceride, UA- uric acid, HDL- high-density lipoprotein cholesterol, LDL- low density lipoprotein cholesterol, SCr- serum creatinine, Ccr- creatinine clearance rate, HR-heart rate, TP-total power of variance, LF-low frequency, HF-high frequency, MetS- metabolic syndrome, PH- Hypertension, DM- Diabetes. FPG and DM duration had 5 missing data, respectively. TG and PBG had 2 missing data, respectively. PH duration has 1 missing data.

### Univariate Logistic Regression Analysis

To estimate the potential risk factors of CA dysfunction, univariate LR analysis was performed in the entire sample, including the demographic parameters, blood glucose, and insulin function parameters; lipid profiles; and medical history factors. The result indicated that 14 potential risk factors–age, HR, BMI, WC, SBP, DBP, FPG, PBG, IR, TG, DM and its duration, and PH and its duration–were significantly associated with CA dysfunction (P<0.05 for all parameters; Table 2).

**Table 2:** Univariate analysis for CA dysfunction.

Variables	N	*B*	*P* value	OR (95% CI)
Age	2092	0.428	<0.001	1.53 (1.35–1.75)
HR	2092	0.859	<0.001	2.36 (2.09–2.67)
BMI	2092	0.273	0.001	1.31 (1.13–1.53)
WC	2092	0.510	<0.001	1.67 (1.3–2.14)
SBP	2092	0.018	<0.001	1.02 (1.01–1.02)
DBP	2092	0.019	0.001	1.02 (1.01–1.03)
FPG	2087	0.450	<0.001	1.57 (1.39–1.78)
PBG	2090	0.475	<0.001	1.61 (1.41–1.83)
IR	2087	0.279	<0.001	1.32 (1.20–1.46)
TG	2090	0.336	0.003	1.40 (1.12–1.75)
DM	2092	0.936	<0.001	2.55 (2.00–3.25)
DM duration	2087	0.412	<0.001	1.51 (1.30–1.76)
PH	2092	0.779	<0.001	2.18 (1.74–2.73)
PH duration	2091	0.356	<0.001	1.43 (1.28–1.59)

Note: HR-heart rate, BMI-body mass index, WC-waist circumference, SBP-systolic blood pressure, DBP-diastolic blood pressure, FPG- fasting plasma glucose, PBG- plasma blood glucose, IR-insulin resistance, TG- triglyceride, PH- Hypertension, DM- Diabetes.

### Multiple Logistic Regression Model

In the multivariate regression model, all these significant variables developed from the univariate analysis and variables with clinical significance were used after stepwise backward elimination of the non-significant variables to obtain the final multivariate regression models, which included seven risk factors of age (odds ratio [OR] ranging from 1.41 to 1.58, P<0.001 for all; Table 3), HR (OR ranging from 2.30 to 2.51, P<0.001 for all), PH (OR 1.39, P = 0.026 for model 1), PH duration (OR ranging from 1.21 to 1.26, P<0.05 for respective models), FINS (OR ranging from 0.28 to 0.36, P<0.05 for respective models), IR (OR ranging from 3.03 to 3.90, P<0.001 for all), or WC (OR ranging from 2.30 to 4.42, P<0.001 for all). Plausible interactions were tested but none of these were significant (data not shown). CA dysfunction was more or less likely with increasing age, HR, PH, IR and WC. Converse tendency of outcome with increasing FINS was found. Five final LR models were developed. All the final models included four common risk factors, namely, age, HR, FINS, and IR. The PH duration was included in five models except for model 1, while the WC was involved in all models except for model 2.

**Table 3:** Final models using Multivariate logistic linear analysis for CA dysfunction.

Models	Variables	*β*	*P* value	*OR* (95% CI)
Model1	Age	0.35	<0.001	1.41 (1.20–1.67)
	HR	0.90	<0.001	2.47 (2.14–2.85)
	PH	0.33	0.0260	1.39 (1.04–1.86)
	lnWC	1.40	0.0410	4.06 (1.06–15.51)
	lnFINS	–1.08	<0.001	0.34 (0.20–0.58)
	lnIR	1.14	<0.001	3.12 (1.90–5.14)
	Constant	–7.88	0.0100	
Model2	Age	0.41	<0.001	1.50 (1.27–1.77)
	HR	0.90	<0.001	2.47 (2.14–2.85)
	PHD	0.23	0.0010	1.26 (1.10–1.44)
	lnWC	1.41	0.0370	4.11 (1.09–15.49)
	lnFINS	–1.02	<0.001	0.36 (0.21–0.63)
	lnIR	1.11	<0.001	3.03 (1.83–5.00)
	Constant	–8.04	0.0080	
Model3	Age	0.46	<0.001	1.58 (1.34–1.87)
	HR	0.92	<0.001	2.51 (2.17–2.91)
	PHD	0.23	0.0010	1.26 (1.11–1.45)
	lnFINS	–1.19	<0.001	0.30 (0.18–0.51)
	lnIR	1.30	<0.001	3.68 (2.29–5.92)
	Constant	–1.63	<0.001	
Model4	Age	0.35	<0.001	1.42 (1.21–1.67)
	HR	0.83	<0.001	2.30 (1.99–2.66)
	PHD	0.19	0.0050	1.21 (1.06–1.39)
	lnWC	1.49	0.0260	4.42 (1.2–16.25)
	lnFINS	–1.18	<0.001	0.31 (0.18–0.53)
	lnIR	1.29	<0.001	3.62 (2.21–5.93)
	Constant	–7.99	0.0070	
Model5	Age	0.43	<0.001	1.53 (1.3–1.81)
	HR	0.85	<0.001	2.33 (2.02–2.7)
	PHD	0.21	0.0020	1.24 (1.08–1.42)
	lnWC	0.83	<0.001	2.30 (1.99–2.66)
	lnFINS	–1.27	<0.001	0.28 (0.16–0.49)
	lnIR	1.36	<0.001	3.90 (2.37–6.41)
	Constant	–1.33	0.0030	

Note: HR-heart rate, PH- hypertension, PHD- hypertension duration, WC-waist circumference, FINS-fasting blood insulin, IR-insulin resistance. DM duration had 5 missing data, respectively. PH duration has 1 missing data.

The area under ROC curve ranged from 0.729–0.790 (Table 4). At the respective optimal cutoff points, when applied to the validation sets, the sensitivity and specificity of the LR models were 68.2–84.9% and 60.2–75.3%, respectively. The positive predictive value ranged from 29.2–38.1% and the negative predictive value ranged from 90.7–94.8%.

**Table 4:** Prediction models using multiple logistic regression and artificial neural network.

Model	AUC (95% CI)		Cutoff point		Sensitivity		Specificity		Yuden Index		PPV		NPV		HL statistics		Accuracy	
Multiple Logistic Regression																		
Model1	0.732 (0.670–0.793)	0.224		0.682		0.677		0.359		0.317		0.907		6.723		0.692		
Model2	0.760 (0.698–0.822)		0.215		0.694		0.753		0.447		0.381		0.918		6.550		0.687	
Model3	0.729 (0.670–0.787)		0.146		0.736		0.609		0.345		0.292		0.913		10.25		0.616	
Model4	0.781 (0.722–0.841)		0.139		0.849		0.602		0.451		0.319		0.948		12.834		0.698	
Model5	0.790 (0.737–0.844)		0.152		0.788		0.668		0.456		0.343		0.935		9.867		0.657	
Artificial Neural Network																		
Model1	0.738 (0.667–0.788)		0.234		0.694		0.694		0.388		0.332		0.912		14.64		0.695	
Model2	0.763 (0.704–0.821)		0.229		0.789		0.663		0.452		0.339		0.935		8.143		0.685	
Model3	0.737 (0.657–0.777)		0.216		0.677		0.647		0.324		0.301		0.898		8.421		0.651	
Model4	0.783 (0.726–0.840)		0.227		0.777		0.704		0.481		0.373		0.932		7.424		0.714	
Model5	0.789 (0.715–0.827)		0.175		0.821		0.618		0.439		0.321		0.940		7.196		0.661	

Note: AUC-Area under the receiver-operating curve, PPV = positive predictive value; NPV = negative predictive value. Data from a total of 2092 patients had been used to MLR analysis. Data from a total of 2077 patients had been used to ANN analysis.

### Artificial Neural Network Model

A total of 15 individuals with 14 risk factors developed from univariate analysis had missing data, so that 2077 individuals were available to form the dataset for development of the artificial neural network prediction model. The same exploratory and validation sets used for the multiple LR analysis were applied for the artificial neural network model, and a total of five ANN models were developed. Every trained ANN included 14 input nodes, 18 layer nodes, and 1 output node (Figure 1). For training ANN, 101–112 echoes were performed and the MSE ranged from 0.12–0.13.

The area under ROC curve ranged from 0.738–0.791 (Table 4). At the respective optimal cutoff points, when applied to the validation sets, the sensitivity and specificity of the ANN models were 67.7–82.1% and 64.7–70.4%, respectively. The positive and negative predictive values ranged from 30.1–37.3% and 89.8–94.0%, respectively.

### Comparison between the Two Models

The diagnostic accuracies of the LR and ANN models are compared in Table 4. Prediction models from LR and ANN based on AUC was similar (P>0.05 for all, data not shown). The mean AUC was 0.758 and 0.762 for the LR and ANN models, and the parameter from ANN model was no inferior to LR one (P<0.001; Table 5). The LR models had a mean sensitivity and specificity of 75.0% and 66.2%, respectively. The mean sensitivity and specificity of the ANN models were 75.1% and 66.7%, respectively. These parameters from ANN model were not inferiority to LR ones (P<0.05 for all, Table 5). Similar results were found between the mean PPV and NPV. The 95% CI widths were narrow in parameters of performance of the ANN model than in the LR model. The HL statistics of the prediction model using LR and ANN analysis were <15.0, indicating that these prediction models showed good fit. The mean values of accuracy were 0.670 and 0.681 for prediction models developed using the LR and ANN approaches, respectively, and non-inferiority result was found (P<0.001, Table 5).

**Table 5:** Comparisons between models from Multiple logistic regression and Artificial neural network analysis.

Parameters	Multiple logistic regression model		Artificial neural network model		*P* value
	Mean ± SD	95% CI	Mean ± SD	95% CI	
AUC	0.758±0.028	0.724–0.793	0.762±0.025	0.732–0.793	<0.001
Cut point	0.175±0.041	0.139–0.211	0.216±0.024	0.187–0.246	0.007
Sensitivity	0.750±0.069	0.664–0.836	0.751±0.065	0.667–0.828	0.014
Specificity	0.662±0.061	0.586–0.738	0.665±0.035	0.622–0.709	0.006
Yuden Index	0.412±0.055	0.344–0.480	0.413±0.063	0.334–0.491	0.045
PPV	0.330±0.034	0.289–0.372	0.330±0.026	0.298–0.361	0.016
NPV	0.924±0.017	0.903–0.945	0.924±0.018	0.902–0.945	<0.001
HL statistics	9.245±2.641	5.966–12.524	9.165±3.103	5.313–13.017	0.246
Accuracy	0.670±0.0340	0.628–0.712	0.681±0.026	0.650–0.713	<0.001

Note: Comparison analysis to parameters of LR and ANN models used noninferiority tests; the null hypothesis was parameters of ANN model were inferior to parameters of LR model (as reference). AUC-Area under the receiver-operating curve, PPV = positive predictive value; NPV = negative predictive value.

## Discussion

We conducted a study to develop and compare prediction models developed using LR and ANN analyses based on a dataset obtained from a large-scale population-based cross-sectional study. The database consisted of 2,092 participants from the Chinese population. The average age of the total sample was >60 years. The prevalence of CA dysfunction in the general population was 18.51%. The participants were a good representative sample across the country, and the prediction model developed in this study might work well even outside the studied areas in China. The prediction model was developed in the exploratory set and was tested in the validation set, and the performance of the developed model was compared between the sets.

Currently, LR and ANN are the most widely used models in biomedicine [8], [14], [19]. LR can generate excellent models and can serve as a commonly accepted statistical tool. Its popularity may be attributed to the interpretability of model parameters and its ease of use. The LR model uses linear combinations of variables, so it is not adept at modeling grossly nonlinear complex interactions, as demonstrated in previous studies on biological and complex epidemiological systems [11]. ANNs are flexible nonlinear systems, and therefore they may be better suited than LR-based models to predict outcomes when the relationships between the variables are complex, multidimensional, and nonlinear, such as those encountered in complex biological systems [10].

The advantages and disadvantages of both the models can be classified according to the following criteria [20]. First, development of an ANN model would require less domain knowledge than that required to develop an LR model. ANNs are ideally suited to modeling complex or unclear relationships since no prior knowledge of the underlying data is required, while the LR model can incorporate complex relationships only if they are explicitly identified. ANNs therefore can model any implicit interactions among input variables commonly encountered in medical data, while LR models are limited as they usually consider only up to two-way interactions. LR models are, in general, less prone to overfitting than ANNs, due to the presence of simpler relationships between the outcome and predictor variables in the former. Development of an LR model requires less computation time than an ANN model. LR models have a distinct advantage over ANNs in existing models developed by other researchers. It is easier to generate confidence intervals in LR models to perform area estimates of risk. LR models have better clinical or real-life inferences than ANNs. ANN parameters do not carry any real-life interpretation; therefore, these models are commonly called black boxes. Varying performance results for LR models versus ANNs have been reported.

Each model has its advantages, and the selection of a model should be based on these advantages and the intended purpose of the study. ANNs would be particularly useful when there are implicit interactions and complex relationships in the data, whereas LR models would be a better choice when statistical inferences have to be drawn from the output. In clinical practice, neither model can replace the other, but the two may be used complementarily to aid in decision making. Both models have the potential to help physicians with respect to understanding CA dysfunction risk factors and diagnosis.

The important finding of this study was that the prediction models developed using LR and ANN analyses have high value in predicting CA dysfunction in the general population. The mean AUCs were 0.758 and 0.762 for LR and ANN analysis, respectively. The mean sensitivity of both the models was >75%. Furthermore, the mean specificity of the two models was >65%. These models were good-fit models based on the large-scale dataset. These findings support that both class models have high predictive value and can be applied to clinical decision making. In this study, prediction models developed using LR and ANN approaches yielded similar performance results. The mean AUCs for LR and ANN analysis showed no significant difference (P = 0.812). The mean sensitivity, specificity, and positive and negative predictive values also showed no significant difference between the two types of models (P>0.05 for all). The parameters of calibration were also similar. These findings support the fact that the predictive ability of the ANN model was comparable to that of the LR model.

These findings should be reproducible in other populations. This and similar models may emerge to be of considerable practical value in patient triage. Suitable ANN software should be designed for clinical practice. LR analysis remains to be the clear choice when the primary goal of model development is to examine possible causal relationships among variables. However, building an ANN or another hybrid technique that incorporates the best features of both the LR and ANN models might result in the development of the ideal prediction model for CA dysfunction.

This study has several limitations. First, the dataset was based on a cross-sectional study and could have been biased by selection. Thus, the temporal sequence between risk factors and outcome was questionable. Second, participants were recruited from Shanghai and external validation was not performed. Therefore, further investigation is required to determine the generalizability of our prediction model. Third, the association between HbAlc was not analyzed in the present study, because data on HbAlc levels were unavailable. Finally, it is important to mention that our study was performed on the Chinese population, and our findings may not be relevant to people of other ethnicities.

In conclusion, this study developed and compared models for the prediction of CA dysfunction in a general Chinese population by using a cross-sectional dataset that was applied to LR and ANN analyses. The predictive ability of the ANN model was comparable to that of the LR model in our dataset. It is necessary to validate the performance of prediction models in an external validation set. A larger and more complete database may be used to further clarify the differences between the ANN and LR models in terms of prediction of the clinical outcome following CA dysfunction.
