# Correction: Comparison of Prediction Model for Cardiovascular Autonomic Dysfunction Using Artificial Neural Network and Logistic Regression Analysis

**DOI:** 10.1371/journal.pone.0176771

**Published:** 2017-04-25

**Authors:** Zi-Hui Tang, Juanmei Liu, Fangfang Zeng, Zhongtao Li, Xiaoling Yu, Linuo Zhou

Prior to publication of this *PLOS ONE* article [1], the authors published closely related work at *BMC Medical Informatics and Decision Making*, in a publication titled "Artificial neural network models for prediction of cardiovascular autonomic dysfunction in general Chinese population" [2]. The journal office has been alerted to concerns regarding the extent of overlap between these two articles.

The authors have explained that the *BMC Medical Informatics and Decision Making* article described work on models based on artificial neural network for the prediction of cardiovascular autonomic dysfunction, while the *PLOS ONE* article reports the comparison between artificial neural network and linear regression models in the same Chinese sample.

The authors wish to make readers aware of the instances of overlap in content between the two articles.

The two articles report analyses on the same dataset, and Table 1, Table 2, and Figure 1 were originally reported in the *BMC Medical Informatics and Decision Making* article. Several sections of the *PLOS ONE* article reused text from the *BMC Medical Informatics and Decision Making* article. In the *PLOS ONE* article, reused text comprises the majority of the Introduction, all Methods subsections except “Multivariate logistic regression models,” and the following text in the Results and Discussion sections:

Results (paragraph 1, “Univariate logistic regression analysis” and “Artificial neural network model” sections)

- “mean FPG, TC, and TG levels […] significantly associated with CA dysfunction”
- “A total of 15 individuals with 14 risk factors […] positive and negative predictive values ranged from 30”
- “statistics of the prediction model using […] developed using the LR and ANN approaches”

Discussion

- “We conducted a study […] no prior knowledge of the underlying data is required.”
- “its advantages, and the selection […] complex relationships in the data”
- “may be used complementarily […] and can be applied to clinical decision making”
- “However, building an ANN […] people of other ethnicities.”

The *PLOS ONE* editors consider that the similarities in content and the re-used text, figure, and tables should have been cited and discussed in the *PLOS ONE* article.

The editors also found that the following text excerpts in the *PLOS ONE* article overlap with other published sources:

- “To obtain the connection weights […] error in output” [4]
- “Each mode has its advantages […] risk factors and diagnosis” [5]

During the evaluation of the above concerns, the editors noted that the authors later published a third article [3] that reports multiple logistic regression analyses for cardiovascular autonomic dysfunction on the same sample.

The authors apologize for not declaring the related manuscript at the time of the submission to *PLOS ONE*, as is required by the journal guidelines, for the reuse of previously published text and results in the *PLOS ONE* article, and for not having adequately discussed the relationship between the studies reported in *BMC Medical Informatics and Decision Making* and *PLOS ONE* in the latter publication.
